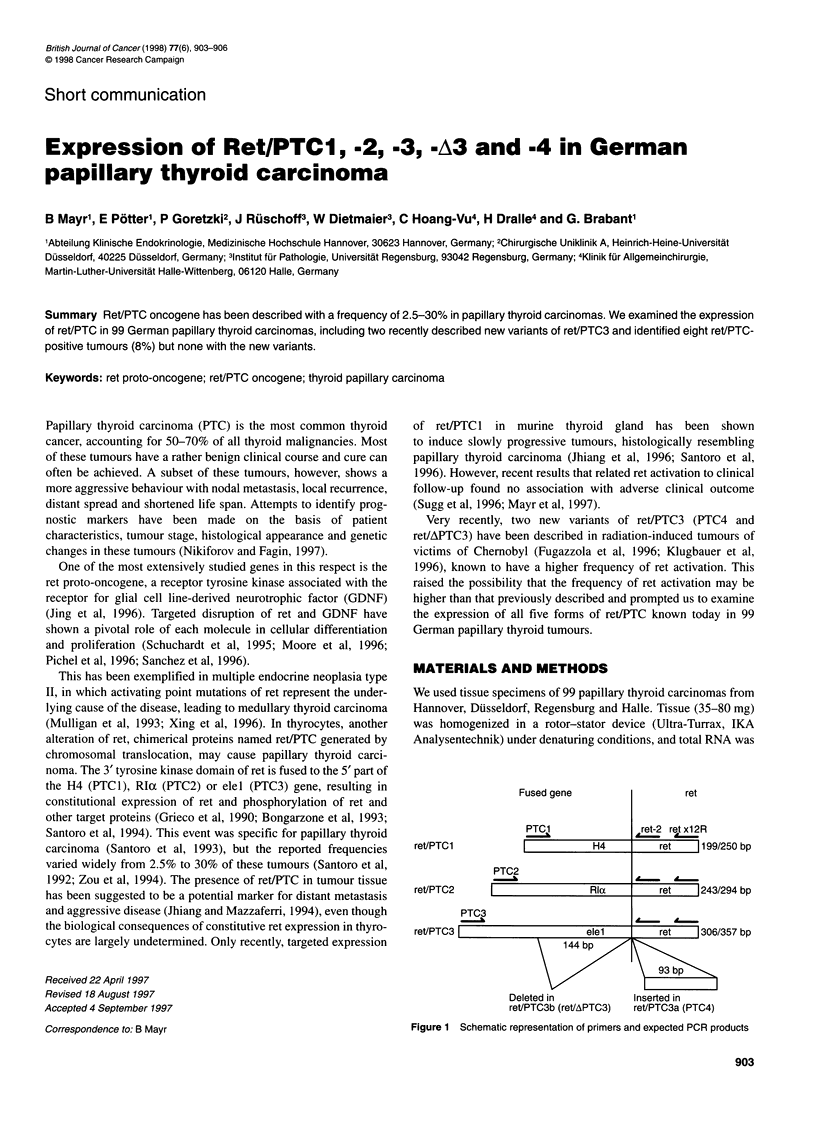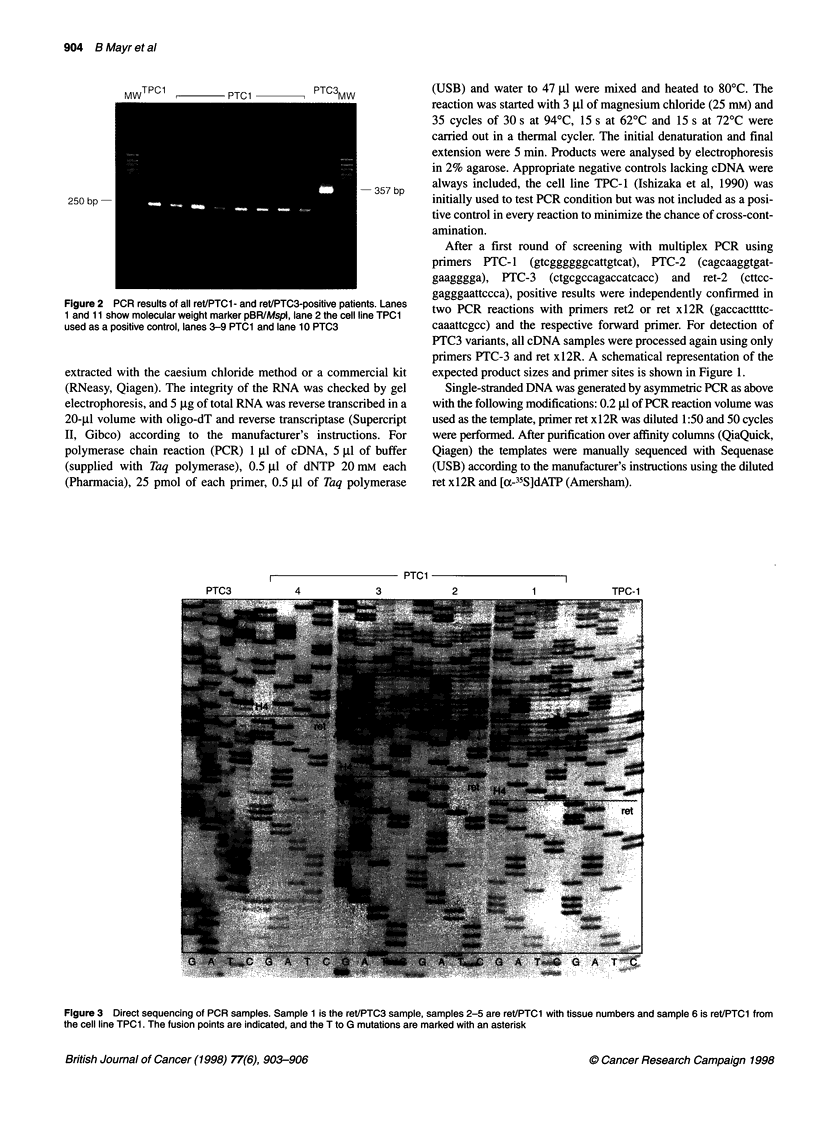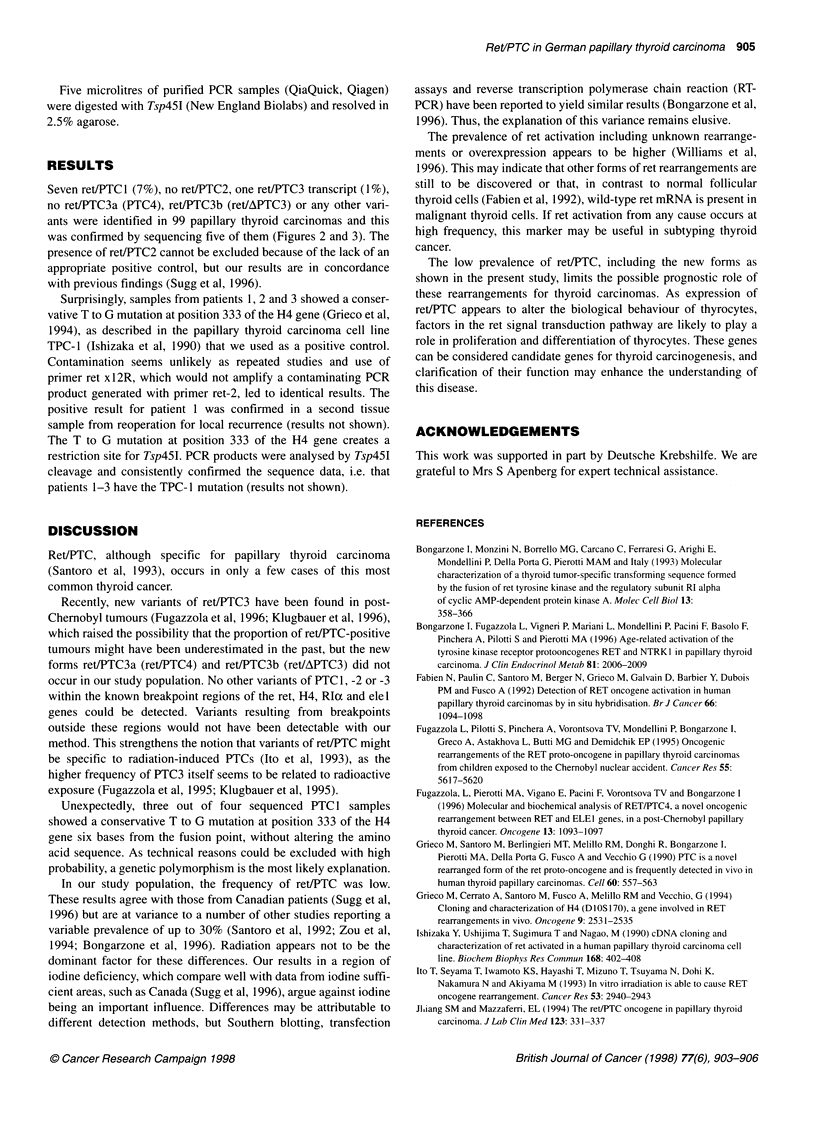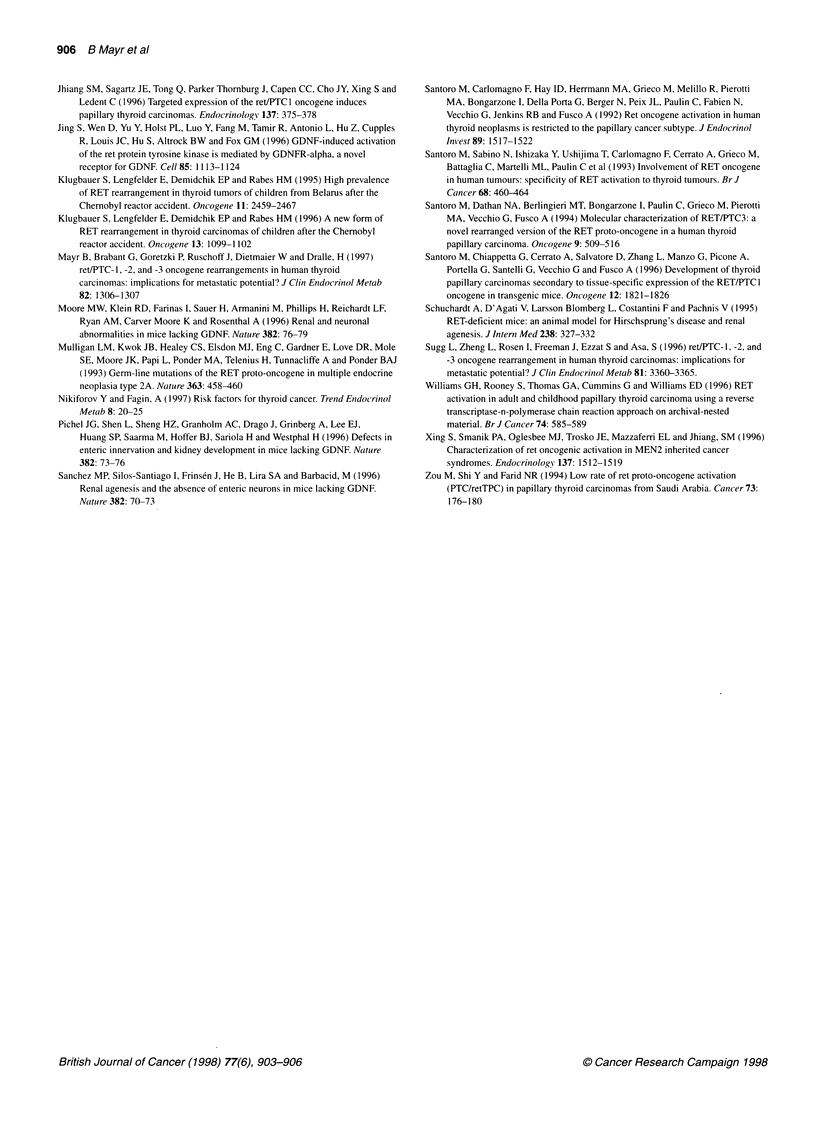# Expression of Ret/PTC1, -2, -3, -delta3 and -4 in German papillary thyroid carcinoma.

**DOI:** 10.1038/bjc.1998.149

**Published:** 1998-03

**Authors:** B. Mayr, E. PÃ¶tter, P. Goretzki, J. RÃ¼schoff, W. Dietmaier, C. Hoang-Vu, H. Dralle, G. Brabant

**Affiliations:** Abteilung Klinische Endokrinologie, Medizinische Hochschule Hannover, Germany.

## Abstract

**Images:**


British Journal of Cancer (1998) 77(6), 903–906
© 1998 Cancer Research Campaign

Short communication

### Expression of Ret/PTC1, -2, -3, -Δ3 and -4 in German papillary thyroid carcinoma

B Mayr[1], E Pötter[1], P Goretzki[2], J Rüschoff[3], W Dietmaier[3], C Hoang-Vu[4], H Dralle[4] and G. Brabant[1]

[1]Abteilung Klinische Endokrinologie, Medizinische Hochschule Hannover, 30623 Hannover, Germany; [2]Chirurgische Uniklinik A, Heinrich-Heine-Universität Düsseldorf, 40225 Düsseldorf, Germany; [3]Institut für Pathologie, Universität Regensburg, 93042 Regensburg, Germany; [4]Klinik für Allgemeinchirurgie, Martin-Luther-Universität Halle-Wittenberg, 06120 Halle, Germany

**Summary** Ret/PTC oncogene has been described with a frequency of 2.5–30% in papillary thyroid carcinomas. We examined the expression of ret/PTC in 99 German papillary thyroid carcinomas, including two recently described new variants of ret/PTC3 and identified eight ret/PTC-positive tumours (8%) but none with the new variants.

**Keywords:** ret proto-oncogene; ret/PTC oncogene; thyroid papillary carcinoma

Papillary thyroid carcinoma (PTC) is the most common thyroid cancer, accounting for 50–70% of all thyroid malignancies. Most of these tumours have a rather benign clinical course and cure can often be achieved. A subset of these tumours, however, shows a more aggressive behaviour with nodal metastasis, local recurrence, distant spread and shortened life span. Attempts to identify prognostic markers have been made on the basis of patient characteristics, tumour stage, histological appearance and genetic changes in these tumours (Nikiforov and Fagin, 1997).

One of the most extensively studied genes in this respect is the ret proto-oncogene, a receptor tyrosine kinase associated with the receptor for glial cell line-derived neurotrophic factor (GDNF) (Jing et al, 1996). Targeted disruption of ret and GDNF have shown a pivotal role of each molecule in cellular differentiation and proliferation (Schuchardt et al, 1995; Moore et al, 1996; Pichel et al, 1996; Sanchez et al, 1996).

This has been exemplified in multiple endocrine neoplasia type II, in which activating point mutations of ret represent the underlying cause of the disease, leading to medullary thyroid carcinoma (Mulligan et al, 1993; Xing et al, 1996). In thyrocytes, another alteration of ret, chimerical proteins named ret/PTC generated by chromosomal translocation, may cause papillary thyroid carcinoma. The 3′ tyrosine kinase domain of ret is fused to the 5′ part of the H4 (PTC1), RIα (PTC2) or ele1 (PTC3) gene, resulting in constitutional expression of ret and phosphorylation of ret and other target proteins (Grieco et al, 1990; Bongarzone et al, 1993; Santoro et al, 1994). This event was specific for papillary thyroid carcinoma (Santoro et al, 1993), but the reported frequencies varied widely from 2.5% to 30% of these tumours (Santoro et al, 1992; Zou et al, 1994). The presence of ret/PTC in tumour tissue has been suggested to be a potential marker for distant metastasis and aggressive disease (Jhiang and Mazzaferri, 1994), even though the biological consequences of constitutive ret expression in thyrocytes are largely undetermined. Only recently, targeted expression of ret/PTC1 in murine thyroid gland has been shown to induce slowly progressive tumours, histologically resembling papillary thyroid carcinoma (Jhiang et al, 1996; Santoro et al, 1996). However, recent results that related ret activation to clinical follow-up found no association with adverse clinical outcome (Sugg et al, 1996; Mayr et al, 1997).

Very recently, two new variants of ret/PTC3 (PTC4 and ret/ΔPTC3) have been described in radiation-induced tumours of victims of Chernobyl (Fugazzola et al, 1996; Klugbauer et al, 1996), known to have a higher frequency of ret activation. This raised the possibility that the frequency of ret activation may be higher than that previously described and prompted us to examine the expression of all five forms of ret/PTC known today in 99 German papillary thyroid tumours.

### MATERIALS AND METHODS

We used tissue specimens of 99 papillary thyroid carcinomas from Hannover, Düsseldorf, Regensburg and Halle. Tissue (35–80 mg) was homogenized in a rotor–stator device (Ultra-Turrax, IKA Analysentechnik) under denaturing conditions, and total RNA was

**Figure 1** Schematic representation of primers and expected PCR products

Received 22 April 1997
Revised 18 August 1997
Accepted 4 September 1997

Correspondence to: B Mayr

**Figure 2** PCR results of all ret/PTC1- and ret/PTC3-positive patients. Lanes 1 and 11 show molecular weight marker pBR/*Msp*I, lane 2 the cell line TPC1 used as a positive control, lanes 3–9 PTC1 and lane 10 PTC3

extracted with the caesium chloride method or a commercial kit (RNeasy, Qiagen). The integrity of the RNA was checked by gel electrophoresis, and 5 μg of total RNA was reverse transcribed in a 20-μl volume with oligo-dT and reverse transcriptase (Supercript II, Gibco) according to the manufacturer's instructions. For polymerase chain reaction (PCR) 1 μl of cDNA, 5 μl of buffer (supplied with *Taq* polymerase), 0.5 μl of dNTP 20 mM each (Pharmacia), 25 pmol of each primer, 0.5 μl of *Taq* polymerase (USB) and water to 47 μl were mixed and heated to 80°C. The reaction was started with 3 μl of magnesium chloride (25 mM) and 35 cycles of 30 s at 94°C, 15 s at 62°C and 15 s at 72°C were carried out in a thermal cycler. The initial denaturation and final extension were 5 min. Products were analysed by electrophoresis in 2% agarose. Appropriate negative controls lacking cDNA were always included, the cell line TPC-1 (Ishizaka et al, 1990) was initially used to test PCR condition but was not included as a positive control in every reaction to minimize the chance of cross-contamination.

After a first round of screening with multiplex PCR using primers PTC-1 (gtcggggggcattgtcat), PTC-2 (cagcaaggtgatgaagggga), PTC-3 (ctgcgccagaccatcacc) and ret-2 (cttccgagggaattccca), positive results were independently confirmed in two PCR reactions with primers ret2 or ret x12R (gaccacttttccaaattcgcc) and the respective forward primer. For detection of PTC3 variants, all cDNA samples were processed again using only primers PTC-3 and ret x12R. A schematical representation of the expected product sizes and primer sites is shown in Figure 1.

Single-stranded DNA was generated by asymmetric PCR as above with the following modifications: 0.2 μl of PCR reaction volume was used as the template, primer ret x12R was diluted 1:50 and 50 cycles were performed. After purification over affinity columns (QiaQuick, Qiagen) the templates were manually sequenced with Sequenase (USB) according to the manufacturer's instructions using the diluted ret x12R and [α-$^{35}$S]dATP (Amersham).

**Figure 3** Direct sequencing of PCR samples. Sample 1 is the ret/PTC3 sample, samples 2–5 are ret/PTC1 with tissue numbers and sample 6 is ret/PTC1 from the cell line TPC1. The fusion points are indicated, and the T to G mutations are marked with an asterisk

© Cancer Research Campaign 1998

Five microlitres of purified PCR samples (QiaQuick, Qiagen) were digested with *Tsp*45I (New England Biolabs) and resolved in 2.5% agarose.

### RESULTS

Seven ret/PTC1 (7%), no ret/PTC2, one ret/PTC3 transcript (1%), no ret/PTC3a (PTC4), ret/PTC3b (ret/ΔPTC3) or any other variants were identified in 99 papillary thyroid carcinomas and this was confirmed by sequencing five of them (Figures 2 and 3). The presence of ret/PTC2 cannot be excluded because of the lack of an appropriate positive control, but our results are in concordance with previous findings (Sugg et al, 1996).

Surprisingly, samples from patients 1, 2 and 3 showed a conservative T to G mutation at position 333 of the H4 gene (Grieco et al, 1994), as described in the papillary thyroid carcinoma cell line TPC-1 (Ishizaka et al, 1990) that we used as a positive control. Contamination seems unlikely as repeated studies and use of primer ret x12R, which would not amplify a contaminating PCR product generated with primer ret-2, led to identical results. The positive result for patient 1 was confirmed in a second tissue sample from reoperation for local recurrence (results not shown). The T to G mutation at position 333 of the H4 gene creates a restriction site for *Tsp*45I. PCR products were analysed by *Tsp*45I cleavage and consistently confirmed the sequence data, i.e. that patients 1–3 have the TPC-1 mutation (results not shown).

### DISCUSSION

Ret/PTC, although specific for papillary thyroid carcinoma (Santoro et al, 1993), occurs in only a few cases of this most common thyroid cancer.

Recently, new variants of ret/PTC3 have been found in post-Chernobyl tumours (Fugazzola et al, 1996; Klugbauer et al, 1996), which raised the possibility that the proportion of ret/PTC-positive tumours might have been underestimated in the past, but the new forms ret/PTC3a (ret/PTC4) and ret/PTC3b (ret/ΔPTC3) did not occur in our study population. No other variants of PTC1, -2 or -3 within the known breakpoint regions of the ret, H4, RIα and ele1 genes could be detected. Variants resulting from breakpoints outside these regions would not have been detectable with our method. This strengthens the notion that variants of ret/PTC might be specific to radiation-induced PTCs (Ito et al, 1993), as the higher frequency of PTC3 itself seems to be related to radioactive exposure (Fugazzola et al, 1995; Klugbauer et al, 1995).

Unexpectedly, three out of four sequenced PTC1 samples showed a conservative T to G mutation at position 333 of the H4 gene six bases from the fusion point, without altering the amino acid sequence. As technical reasons could be excluded with high probability, a genetic polymorphism is the most likely explanation.

In our study population, the frequency of ret/PTC was low. These results agree with those from Canadian patients (Sugg et al, 1996) but are at variance to a number of other studies reporting a variable prevalence of up to 30% (Santoro et al, 1992; Zou et al, 1994; Bongarzone et al, 1996). Radiation appears not to be the dominant factor for these differences. Our results in a region of iodine deficiency, which compare well with data from iodine sufficient areas, such as Canada (Sugg et al, 1996), argue against iodine being an important influence. Differences may be attributable to different detection methods, but Southern blotting, transfection assays and reverse transcription polymerase chain reaction (RT-PCR) have been reported to yield similar results (Bongarzone et al, 1996). Thus, the explanation of this variance remains elusive.

The prevalence of ret activation including unknown rearrangements or overexpression appears to be higher (Williams et al, 1996). This may indicate that other forms of ret rearrangements are still to be discovered or that, in contrast to normal follicular thyroid cells (Fabien et al, 1992), wild-type ret mRNA is present in malignant thyroid cells. If ret activation from any cause occurs at high frequency, this marker may be useful in subtyping thyroid cancer.

The low prevalence of ret/PTC, including the new forms as shown in the present study, limits the possible prognostic role of these rearrangements for thyroid carcinomas. As expression of ret/PTC appears to alter the biological behaviour of thyrocytes, factors in the ret signal transduction pathway are likely to play a role in proliferation and differentiation of thyrocytes. These genes can be considered candidate genes for thyroid carcinogenesis, and clarification of their function may enhance the understanding of this disease.

### ACKNOWLEDGEMENTS

This work was supported in part by Deutsche Krebshilfe. We are grateful to Mrs S Apenberg for expert technical assistance.

### REFERENCES

Bongarzone I, Monzini N, Borrello MG, Carcano C, Ferraresi G, Arighi E, Mondellini P, Della Porta G, Pierotti MAM and Italy (1993) Molecular characterization of a thyroid tumor-specific transforming sequence formed by the fusion of ret tyrosine kinase and the regulatory subunit RI alpha of cyclic AMP-dependent protein kinase A. *Molec Cell Biol* **13**: 358–366

Bongarzone I, Fugazzola L, Vigneri P, Mariani L, Mondellini P, Pacini F, Basolo F, Pinchera A, Pilotti S and Pierotti MA (1996) Age-related activation of the tyrosine kinase receptor protooncogenes RET and NTRK1 in papillary thyroid carcinoma. *J Clin Endocrinol Metab* **81**: 2006–2009

Fabien N, Paulin C, Santoro M, Berger N, Grieco M, Galvain D, Barbier Y, Dubois PM and Fusco A (1992) Detection of RET oncogene activation in human papillary thyroid carcinomas by in situ hybridisation. *Br J Cancer* **66**: 1094–1098

Fugazzola L, Pilotti S, Pinchera A, Vorontsova TV, Mondellini P, Bongarzone I, Greco A, Astakhova L, Butti MG and Demidchik EP (1995) Oncogenic rearrangements of the RET proto-oncogene in papillary thyroid carcinomas from children exposed to the Chernobyl nuclear accident. *Cancer Res* **55**: 5617–5620

Fugazzola, L, Pierotti MA, Vigano E, Pacini F, Vorontsova TV and Bongarzone I (1996) Molecular and biochemical analysis of RET/PTC4, a novel oncogenic rearrangement between RET and ELE1 genes, in a post-Chernobyl papillary thyroid cancer. *Oncogene* **13**: 1093–1097

Grieco M, Santoro M, Berlingieri MT, Melillo RM, Donghi R, Bongarzone I, Pierotti MA, Della Porta G, Fusco A and Vecchio G (1990) PTC is a novel rearranged form of the ret proto-oncogene and is frequently detected in vivo in human thyroid papillary carcinomas. *Cell* **60**: 557–563

Grieco M, Cerrato A, Santoro M, Fusco A, Melillo RM and Vecchio, G (1994) Cloning and characterization of H4 (D10S170), a gene involved in RET rearrangements in vivo. *Oncogene* **9**: 2531–2535

Ishizaka Y, Ushijima T, Sugimura T and Nagao, M (1990) cDNA cloning and characterization of ret activated in a human papillary thyroid carcinoma cell line. *Biochem Biophys Res Commun* **168**: 402–408

Ito T, Seyama T, Iwamoto KS, Hayashi T, Mizuno T, Tsuyama N, Dohi K, Nakamura N and Akiyama M (1993) In vitro irradiation is able to cause RET oncogene rearrangement. *Cancer Res* **53**: 2940–2943

Jhiang SM and Mazzaferri, EL (1994) The ret/PTC oncogene in papillary thyroid carcinoma. *J Lab Clin Med* **123**: 331–337

© Cancer Research Campaign 1998

Jhiang SM, Sagartz JE, Tong Q, Parker Thornburg J, Capen CC, Cho JY, Xing S and Ledent C (1996) Targeted expression of the ret/PTC1 oncogene induces papillary thyroid carcinomas. *Endocrinology* **137**: 375–378

Jing S, Wen D, Yu Y, Holst PL, Luo Y, Fang M, Tamir R, Antonio L, Hu Z, Cupples R, Louis JC, Hu S, Altrock BW and Fox GM (1996) GDNF-induced activation of the ret protein tyrosine kinase is mediated by GDNFR-alpha, a novel receptor for GDNF. *Cell* **85**: 1113–1124

Klugbauer S, Lengfelder E, Demidchik EP and Rabes HM (1995) High prevalence of RET rearrangement in thyroid tumors of children from Belarus after the Chernobyl reactor accident. *Oncogene* **11**: 2459–2467

Klugbauer S, Lengfelder E, Demidchik EP and Rabes HM (1996) A new form of RET rearrangement in thyroid carcinomas of children after the Chernobyl reactor accident. *Oncogene* **13**: 1099–1102

Mayr B, Brabant G, Goretzki P, Ruschoff J, Dietmaier W and Dralle, H (1997) ret/PTC-1, -2, and -3 oncogene rearrangements in human thyroid carcinomas: implications for metastatic potential? *J Clin Endocrinol Metab* **82**: 1306–1307

Moore MW, Klein RD, Farinas I, Sauer H, Armanini M, Phillips H, Reichardt LF, Ryan AM, Carver Moore K and Rosenthal A (1996) Renal and neuronal abnormalities in mice lacking GDNF. *Nature* **382**: 76–79

Mulligan LM, Kwok JB, Healey CS, Elsdon MJ, Eng C, Gardner E, Love DR, Mole SE, Moore JK, Papi L, Ponder MA, Telenius H, Tunnacliffe A and Ponder BAJ (1993) Germ-line mutations of the RET proto-oncogene in multiple endocrine neoplasia type 2A. *Nature* **363**: 458–460

Nikiforov Y and Fagin, A (1997) Risk factors for thyroid cancer. *Trend Endocrinol Metab* **8**: 20–25

Pichel JG, Shen L, Sheng HZ, Granholm AC, Drago J, Grinberg A, Lee EJ, Huang SP, Saarma M, Hoffer BJ, Sariola H and Westphal H (1996) Defects in enteric innervation and kidney development in mice lacking GDNF. *Nature* **382**: 73–76

Sanchez MP, Silos-Santiago I, Frinsén J, He B, Lira SA and Barbacid, M (1996) Renal agenesis and the absence of enteric neurons in mice lacking GDNF. *Nature* **382**: 70–73

Santoro M, Carlomagno F, Hay ID, Herrmann MA, Grieco M, Melillo R, Pierotti MA, Bongarzone I, Della Porta G, Berger N, Peix JL, Paulin C, Fabien N, Vecchio G, Jenkins RB and Fusco A (1992) Ret oncogene activation in human thyroid neoplasms is restricted to the papillary cancer subtype. *J Endocrinol Invest* **89**: 1517–1522

Santoro M, Sabino N, Ishizaka Y, Ushijima T, Carlomagno F, Cerrato A, Grieco M, Battaglia C, Martelli ML, Paulin C et al (1993) Involvement of RET oncogene in human tumours: specificity of RET activation to thyroid tumours. *Br J Cancer* **68**: 460–464

Santoro M, Dathan NA, Berlingieri MT, Bongarzone I, Paulin C, Grieco M, Pierotti MA, Vecchio G, Fusco A (1994) Molecular characterization of RET/PTC3: a novel rearranged version of the RET proto-oncogene in a human thyroid papillary carcinoma. *Oncogene* **9**: 509–516

Santoro M, Chiappetta G, Cerrato A, Salvatore D, Zhang L, Manzo G, Picone A, Portella G, Santelli G, Vecchio G and Fusco A (1996) Development of thyroid papillary carcinomas secondary to tissue-specific expression of the RET/PTC1 oncogene in transgenic mice. *Oncogene* **12**: 1821–1826

Schuchardt A, D'Agati V, Larsson Blomberg L, Costantini F and Pachnis V (1995) RET-deficient mice: an animal model for Hirschsprung's disease and renal agenesis. *J Intern Med* **238**: 327–332

Sugg L, Zheng L, Rosen I, Freeman J, Ezzat S and Asa, S (1996) ret/PTC-1, -2, and -3 oncogene rearrangement in human thyroid carcinomas: implications for metastatic potential? *J Clin Endocrinol Metab* **81**: 3360–3365.

Williams GH, Rooney S, Thomas GA, Cummins G and Williams ED (1996) RET activation in adult and childhood papillary thyroid carcinoma using a reverse transcriptase-n-polymerase chain reaction approach on archival-nested material. *Br J Cancer* **74**: 585–589

Xing S, Smanik PA, Oglesbee MJ, Trosko JE, Mazzaferri EL and Jhiang, SM (1996) Characterization of ret oncogenic activation in MEN2 inherited cancer syndromes. *Endocrinology* **137**: 1512–1519

Zou M, Shi Y and Farid NR (1994) Low rate of ret proto-oncogene activation (PTC/retTPC) in papillary thyroid carcinomas from Saudi Arabia. *Cancer* **73**: 176–180

© Cancer Research Campaign 1998